# Programmed Cell Death Ligand 1 Is Enriched in Mammary Stem Cells and Promotes Mammary Development and Regeneration

**DOI:** 10.3389/fcell.2021.772669

**Published:** 2021-11-05

**Authors:** Ruirui Wang, Fujing Huang, Wei Wei, Yu Zhou, Zi Ye, Liya Yu, Junyuan Hu, Cheguo Cai

**Affiliations:** ^1^Department of Orthopaedics, Zhongnan Hospital of Wuhan University, Frontier Science Center for Immunology and Metabolism, Medical Research Institute, Wuhan University, Wuhan, China; ^2^Shenzhen Beike Biotechnology Co., Ltd., Shenzhen, China; ^3^Dongguan and Guangzhou University of Chinese Medicine Cooperative Academy of Mathematical Engineering for Chinese Medicine, Dongguan, China

**Keywords:** PD-L1, mammary stem cell, mammary regeneration, mammary development, cross-talk, niche

## Abstract

Programmed cell death ligand 1 (PD-L1) is widely expressed in a variety of human tumors, and inhibition of the PD-L1/PD-1 pathway represents one of the most promising therapy for many types of cancer. However, the physiological function of PD-L1 in tissue development is still unclear, although *PD-L1* mRNA is abundant in many tissues. To address this puzzle, we investigated the function of PD-L1 in mammary gland development. Interestingly, we found that PD-L1 is enriched in protein C receptor (Procr)-expressing mammary stem cells (MaSCs), and PD-L1-expressing mammary basal cells (PD-L1^+^ basal cells) exhibit robust mammary regeneration capacity in transplantation assay. The lineage tracing experiment showed that PD-L1^+^ cells can differentiate into all lineages of mammary epithelium cells, suggesting that PD-L1^+^ basal cells have the activities of MaSCs. Furthermore, *PD-L1* deficiency significantly impairs mammary development and reduces mammary regeneration capacity of mammary basal cells, suggesting that *PD-L1* is not only enriched in MaSCs but also improves activities of MaSCs. In summary, these results demonstrated that *PD-L1* is enriched in MaSCs and promotes mammary gland development and regeneration. Mechanistically, our data indicated that *PD-L1* expression is induced by continuous activation of Wnt/ß-catenin signaling. In conclusion, these results demonstrated that PD-L1 is a marker of MaSCs, and *PD-L1* is essential for mammary development. Our study provides novel insight into the physiological functions of PD-L1 in tissue development.

## Introduction

Programmed cell death ligand 1 (PD-L1 or B7-H1) is a member of the B7 immunoglobulin superfamily (Dong et al., 1999). PD-L1 is known to be expressed on tumor cells and/or tumor infiltration immune cells, and its interaction with PD-1 expressed on activated T cells inhibits the T cell responses against the tumor, enabling tumor immune evasion (Dong et al., 2002; Chen, 2004; Zou and Chen, 2008; Pardoll, 2012; Kamphorst et al., 2017). Pharmacological blockade of the PD-1/PD-L1 interaction restores the anti-tumor immune response and represents one of the most promising therapy against cancer (Pardoll, 2012; Iwai et al., 2017; Sharpe and Pauken, 2018). To date, inhibition of the PD-1/PD-L1 pathway by antibodies has become a very powerful therapeutic strategy for patients with various types of cancer and has shown unprecedented clinical responses in advanced tumors (Alsaab et al., 2017).

Under physiological conditions, the *PD-L1* mRNA is abundant in many tissues and organs of humans and mice (Dong et al., 1999; Freeman et al., 2000; Brown et al., 2003), including both lymphoid and non-lymphoid organs (Yao et al., 2013). However, the PD-L1 protein is only constitutively expressed on immune cells (Dong et al., 1999, 2019; Freeman et al., 2000; Curiel et al., 2003; Latchman et al., 2004; Saudemont et al., 2005; Terme et al., 2012; Iraolagoitia et al., 2016; Hartley et al., 2018) and is inducibly expressed in activated vascular endothelial cells (Mazanet and Hughes, 2002), mesenchymal stem cells (Augello et al., 2005), and cultured bone marrow-derived mast cells (Nakae et al., 2006). However, little is known about the physiological function of PD-L1 in organ development and tissue regeneration.

The mammary gland is a highly regenerative organ (Faulkin and Deome, 1960; Daniel, 1975). It has a unique postnatal development feature and undergoes profound changes within its epithelium during puberty and reproductive cycles (Borellini and Oka, 1989; Inman et al., 2015). At puberty, the ductal epithelium of the mammary anlage invades the mammary fat pad and forms a ductal tree, which is orchestrated by a specialized structure called the terminal end bud (TEB). At the onset of puberty, the TEBs emerge from the stratified epithelium of the immature gland; TEBs are responsible for the production of mature cell types (basal and luminal cells) leading to the elongation of the ducts (Paine and Lewis, 2017). Studies have shown that some degree of epithelial-to-mesenchymal transition (EMT) occurs at the TEB (Kouros-Mehr and Werb, 2006; Nelson et al., 2006). During pregnancy, the glands of virgin adult mice develop a network of milk-secreting alveoli lined by specialized luminal cells. Thus, the formation of pubertal TEBs, the elongation of ducts, and the formation of alveolar during pregnancy are illustrative examples of the regenerative potential of the mammary epithelium and its ability to undergo many cycles of growth and involution.

The regenerative capacity of the mammary gland is mediated by mammary gland stem cells (MaSCs) (Inman et al., 2015). MaSC is a rare population of mammary epithelial cells capable of generating the entire mammary epithelial architecture (Kordon and Smith, 1998; Shackleton et al., 2006; Stingl et al., 2006). Transplantation assays, which have been extensively used to evaluate the regeneration capacity of a different subset of epithelial cells, have demonstrated that MaSCs occur exclusively among mammary basal cells (Shackleton et al., 2006; Stingl et al., 2006; Zeng and Nusse, 2010; Rios et al., 2014). Accordingly, the basal cell-specific proteins keratin14 (K14) and keratin 5 (K5) are used to label MaSCs (Van Keymeulen et al., 2011; Rios et al., 2014). Subsequent studies have demonstrated that Wnt/ß-catenin signaling regulates MaSC self-renewal and have shown that the Lgr5 and Axin2 can be used to more specifically label MaSCs amongst mammary basal cells (Van Keymeulen et al., 2011; de Visser et al., 2012; van Amerongen et al., 2012; Rios et al., 2014). Recently, we found that protein C receptor (Procr), a target gene of Wnt/ß-catenin signaling, marks a population of multipotent MaSCs among the mammary basal cells (Wang et al., 2015). Further studies indicated that Procr is a druggable target on the surface of breast cancer stem cells (CSCs) (Wang et al., 2019), which established a connection between MaSCs in normal tissues and CSCs.

Here, we investigated the physiological roles of PD-L1 in mammary development and regeneration. We found that PD-L1 is enriched in Procr-expressing mammary basal cells, and PD-L1 labeled a subset of mammary basal cells (PD-L1^+^ basal cells). PD-L1^+^ basal cells are able to differentiate into all mammary epithelium cell lineages, suggesting that PD-L1^+^ basal cells have activities of multipotent MaSCs. In transplantation assay, PD-L1^+^ basal cells exhibit excellent regeneration capacity. In addition, *PD-L1* deficiency impaired mammary gland development and regeneration, and overexpression of PD-L1 significantly increased mammary regeneration frequency. Thus, our study showed that PD-L1 regulates the development and regeneration of the mammary gland, which provided new insight into the physiological function of PD-L1.

## Materials and Methods

### Mouse Strains

Programmed cell death ligand 1-CreERT2-2A-tdTomato (C57BL/6J) mice were generated as illustrated in this article in Figure 2, heterozygous mice (*PD-L1*^+/–^) were healthy and fertile, homozygous mice (*PD-L1^–^*^/^*^–^*, *PD-L1* deficiency) were fertile, but were more vulnerable to suffer from autoimmune disease, resembling the *PD-L1*-null mutant mice reported before (Dong et al., 2004); *Rosa26*-mTmG mice were purchased from the Jackson Laboratory. In this study, C57BL/6J mice at 6–8 weeks old were used for primary cell preparation, and nude mice were strain at 3 weeks old for transplantation assay. Experimental procedures were approved by the Animal Care and Use Committee of Wuhan University. All mice used in the transplantation experiments were females.

### Tracing Experiment

Programmed cell death ligand 1-CreERT2-2A-tdTomato/*Rosa26*-mTmG mice were intraperitoneally injected with a certain dose of tamoxifen (TAM; Sigma-Aldrich, Burlington, MA, United States) diluted in corn oil containing 10% ethanol; 4 mg/25 g body weight of TAM was performed to induce mice at puberty. Mammary glands were harvested at different time points followed by fluorescence-activated cell sorting (FACS) analysis and immunostaining.

### Cloning, Viral Production, and Infection

For lentivirus-mediated overexpression studies, the transfer vector plasmid (pWPI) plasmids containing full-length cDNA of *PD-L1* were created by routine molecular cloning techniques, the cDNA of *PD-L1* was cloned by mouse genome cDNA.

All plasmids with helper plasmids pCMV-dR8.9 and pVSV-G (Chen et al., 2005) were packaged into the virus using HEK293-T cells as packaging cell lines following standard protocols. Lentiviruses were collected 48–72 h after transfection.

### Western Blot

Proteins from cells and tissues were extracted using Radio-Immunoprecipitation Assay (RIPA; Sigma, United States) buffer with protease inhibitor Phenylmethanesulfonyl Fluoride (PMSF; Sigma) added, then incubated on ice for 20 min; cell lysates were centrifuged at 10,000 *g* for 5 min at 4°C, and then supernatants obtained were incubated at 99°C for 10 min to denature the proteins. Western blot analysis was conducted following standard protocol. The following antibodies were used: rat anti-mouse PD-L1 (1:1000, R&D, United States, MAB1019) and mouse anti-Actin (1:5000, Sigma, A2228).

### Quantitative Real-Time-PCR Analysis

Total RNA was extracted from sorted mammary epithelial cells using RNA TRIzol reagent (Takara, Japan) according to the instructions of the manufacturer. Reverse transcription was conducted using the cDNA synthesis kit (Takara). Synthetic template cDNA was stored at −20°C for a long time. Quantitative Real Time (RT)-PCR was performed using the FastStart Universal SYBR Green Master Mix kit (Roche, Basel, Switzerland). Relative mRNA levels were normalized to the *GAPDH* mRNA levels.

Quantitative RT-PCR primers used were as follows:

**Table:** 

*GAPDH*-qF	5′-AGGTCGGTGTGAACGGATTTG-3′
*GAPDH*-qR	5′-TGTAGACCATGTAGTTGAGGTCA-3′
*PD-L1*-qF	5′-GCTCCAAAGGACTTGTACGTG-3′
*PD-L1*-qR	5′-TGATCTGAAGGGCAGCATTTC-3
*Procr*-qF	5′-CTCTCTGGGAAAACTCCTGACA-3′
*Procr*-qR	5′-CAGGGAGCAGCTAACA GTGA-3′
*Axin2*-qF	5′-TGACTCTCCTTCCAGATCCCA-3′
*Axin2*-qR	5′-TGCCCACACTAGGCTGACA-3′

### Primary Single-Cell Preparation

Mammary glands of the fourth pad from 6 to 8 weeks old female mice were dissected and isolated. The minced fresh tissue was placed in digestion medium (RPMI 1640 [Hyclone] + 5% fetal bovine serum [PAN Biotech] + 1% penicillin/streptomycin [Hyclone] + 25 mM 4-1-piperazineethanesulfonic acid, HEPES, [Sigma]) with 300 U/ml collagenase III (Worthington, United States) added and digested for 2 h at 37°C, 100 rpm with a good shaking in every 15 min. After lysis of erythrocytes by Red Blood Cell Lysis Buffer (Sigma) for 5 min at room temperature, single-cell suspension was obtained by incubation with 0.05% trypsin-ethylenediaminetetraacetic acid (EDTA; Hyclone) for 5 min at 37°C, then 0.1 mg/ml DNase I (Sigma) for another 5 min at 37°C with pipetting gently, finally followed by filtration through 70 μm cell strainers (Corning, United States), and then the primary cells were well prepared.

### Flow Cytometry Cell Sorting and Antibodies

The following antibodies were used in this study:

Fluorescein isothiocyanate (FITC)-conjugated CD31 (BD Biosciences, United States, 553372), CD45 (BD Biosciences, 553080), TER119 (BD Biosciences, 557915); biotinylated CD31 (BD Biosciences, 553371), CD45 (BD Biosciences, 553078), TER119 (BD Biosciences, 553672); CD24-PE/cy7 (Biolegend, United States, 101822), CD29-APC (Biolegend, 102216), Procr-PE (eBioscience, United States, 12-2012-82), PD-L1-PE (eBioscience, 124308), Streptavidin-APC-eFluor 780 (eBioscience, 47-4317-82), and Streptavidin-V450 (eBioscience, 48-4317-82). Antibody incubation was performed on ice for 20–25 min in PBS with 5% fetal bovine serum. All the antibodies were employed at 1:200 dilutions. Before cell sorting and analysis, cells were filtered through 40 μm cell strainers. Single cells gating and sorting were performed using an FACS BD Aria III or Moflo XDP Beckman flow cytometer. All analyses were performed using an LSRFortessa X20. FACS data were analyzed using FlowJo software (Tree Star, United States).

### Colony Formation Assay

Cells isolated by FACS or infected cells obtained from Ultra-low attached 24 well plated (Corning) were resuspended in 100% growth-factor-reduced Matrigel (Corning), and the gels mixed with target cells were allowed to polymerize before covering with culture medium (F12 [Hyclone], ITS [1:100; Gibco^TM^], 50 ng/ml EGF [Corning]), plus either 200 ng/ml Wnt3aor 3 mM CHIR (Selleck; United States, S1263). The culture medium was changed every 24 h. Primary colony numbers were measured after being cultured for 5–7 days. The colonies were spherical; the long axis was measured in cases that colonies were oval.

### Whole-Mount Analysis

Mammary glands of the fourth pair fat pad from female mice were harvested and cut into 3 mm × 3 mm to be placed in digestion medium (RPMI 1640 + 5% fetal bovine serum + 1% penicillin/streptomycin + 25 mM HEPES) with 300 U/ml collagenase III added and digested for 40 min at 37°C. Dissected mammary glands were fixed in 4% paraformaldehyde for 1 h at 4°C. Fixed tissues were washed three times and then blocked by 1 ml MABT (0.5% Tween-20 in malic acid buffer) for 1 h at room with 10% fetal bovine serum added for 1 h at room temperature. The tissues were incubated with primary antibodies Rabbit anti-Krt 14 (1:250, BioLengend, 90530SH) overnight at 4°C, followed by washes, and then secondary antibody was incubated with Donkey anti-Rabbit 1:500 overnight at 4°C. After washing, the tissues were put into 3 ml 80% sucrose overnight at 4°C with a good shaking. The next day, tissues were mounted with prolong gold antifade reagent with 4′,6-diamidino-2-phenylindole (DAPI; Invitrogen, United States). Tablets with weights for 8–12 h before confocal imaging was performed.

### Mammary Fat Pad Transplantation and Analysis

Sorted cells were pelleted and resuspended in 50% Matrigel, PBS with 50% fetal bovine serum, and 0.04% Trypan Blue (Sigma). Female nude recipient mice at 3 weeks old were anesthetized and a small incision was made to reveal the fourth pair of mammary glands, then a 10 μl volume containing indicated cells was injected into each of the cleared fat pads. Reconstituted mammary glands were harvested and examined 6–8 weeks after surgery. Outgrowth degree of mammary glands was detected under a dissection microscope (Leica, Germany) after carmine staining. For limiting dilution analyses, the repopulating frequency was calculated by the Extreme Limiting Dilution Analysis Program.

### Immunohistochemistry and Antibodies

Frozen sections were hydrated with PBT (0.1% Triton X-100 [Sigma-Aldrich] in PBS) for 10 min at room temperature. Sections were blocked in blocking buffer (10% goat serum [Gibco^TM^] + 0.1% Triton X-100 in PBS) for 2 h at room temperature and then incubated with primary antibodies Rabbit anti-Krt 14 (1:500, BioLegend, 905304), Rat anti-Krt 8 (1:500, DSHB, TROMA-I) at 4°C overnight, followed by washes. Secondary antibodies were incubated for 1 h at room temperature. After washing, sections were mounted with prolong gold antifade reagent with DAPI (Invitrogen).

### Confocal Microscopy

Confocal images of whole-mount and sections were acquired using a Zeiss LSM 880 confocal microscope (Germany). The images obtained were processed with the ZEN v3.1 software.

### Smart-Seq II and Data Analysis

Programmed cell death ligand plus basal cells and PD-L1^–^ basal cells were FACS-isolated from the fourth pair mammary glands of 8 weeks old C57BL/6J mice. Samples according to the instruction manual of the TRIzol reagent. High-quality RNA was sent to Wuhan Bioacme Biological Technologies Corporation (Wuhan, China) for cDNA libraries construction and sequencing. RNA sequencing libraries were generated using the KAPA Stranded RNA-Seq Kit for Illumina with multiplexing primers, according to the manufacturer’s protocol. Then sequencing was performed on Illumina Nova sequencer. The number of perfect clean tags for each gene was calculated and then normalized in fragments per kilo bases per million mapped reads (FPKM) using the described method. Differential expression analysis of two groups was performed using the DESeq R package (1.10.1). The resulting *P*-values were adjusted using Benjamini and Hochberg’s approach for controlling the false discovery rate. Genes with an adjusted *P*-value < 0.05 found by DESeq were assigned as differentially expressed. A heatmap of a collection of genes was generated by Helm software.

### Gene Set Enrichment Analysis

Gene set enrichment analysis (GSEA) v4.1.0 was used to perform the GSEA on various characteristic gene signatures. Normalized microarray expression data were rank ordered by differential expression between cell populations and/or genetic background as indicated, using the provided ratio of classes (i.e., fold change) metric. The MaSC gene signatures were defined by significantly upregulated genes (*P* < 0.05 and *FC* > 3) in cells with high expression of stem cell markers compared with those with low expression of stem cell markers.

### Quantification and Statistical Analysis

Statistical analysis was performed using GraphPad Prism 7.0. For all figure legends with error bars, the SD was calculated to indicate the variation within each experiment. The Student’s *t*-test was evaluated for two pair comparisons. All the experiments have been tested on the basis of three independent experiments unless otherwise specified. Statistical data were considered significant if *P* < 0.05.

## Results

### *PD-L1* Is Enriched in Procr-Expressing Mammary Basal Cells

**FIGURE 1:**
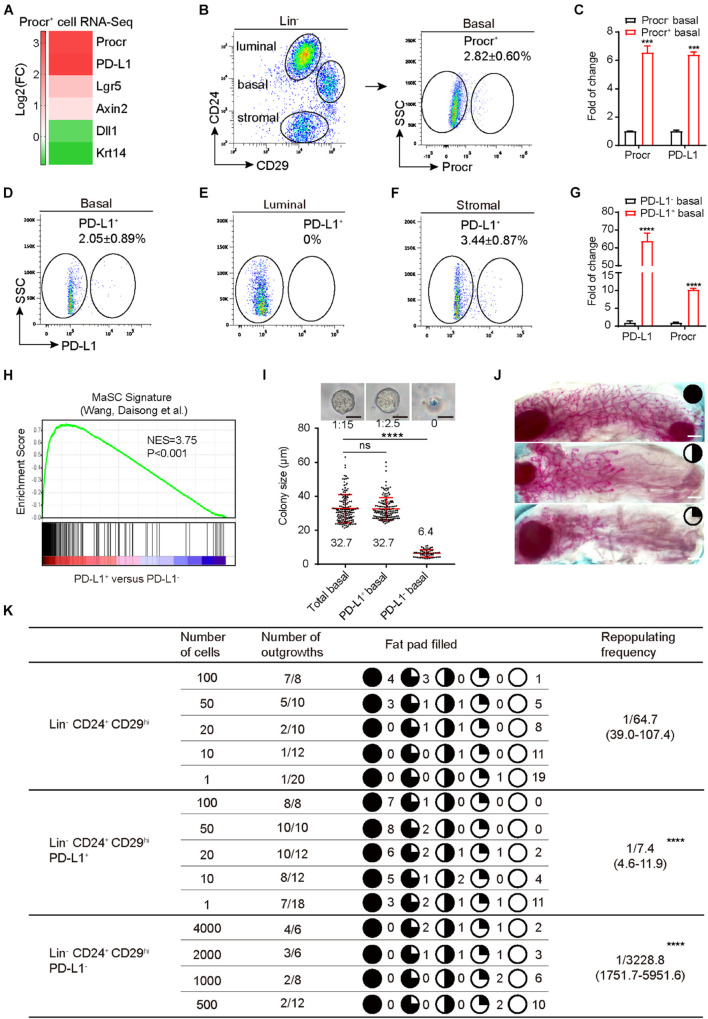
PD-L1 labels a subset of mammary basal cells with high regeneration capacity. **(A)** PD-L1 expression was enriched in Procr^+^ MaSCs (Procr^+^ mammary basal cells). Heat map analysis for Procr^+^ MaSCs compared with Procr^–^ basal cells. **(B)** Isolation of Procr^+^ MaSCs by FACS. **(C)** Real-time quantification PCR (qPCR) analysis of *PD-L1* mRNA levels in Procr^+^ basal and Procr^–^ basal cells, with *GAPDH* as an internal control. Data are presented as mean ± SD. Unpaired *t*-test: *** *P* < 0.001; *n* = 3 mice. **(D–F)** FACS analysis of PD-L1 expression in mammary glands of adult mice. PD-L1^+^ cells were distributed among basal cells (2.05 ± 0.89%) **(D)**, and stromal cells (3.44 ± 0.87%) **(F)**, no PD-L1^+^ cells were detected among the luminal cells **(E)**; *n* = 3 mice. **(G)** Real-time quantification PCR (qPCR) analysis of *Procr* mRNA levels in PD-L1^+^ basal and PD-L1*^–^* basal cells, with *GAPDH* as an internal control. Data are presented as mean ± SD. Unpaired *t*-test: **** *P* < 0.0001; *n* = 3 mice. **(H)** GSEA demonstrating enriched gene signatures of Procr^+^ MaSC in the PD-L1^+^ basal cells as compared to the PD-L1*^–^* basal cells. **(I)** Colony formation efficiency and colony size of total (Lin^–^ CD24^+^ CD29^hi^), PD-L1^+^ (Lin*^–^* CD24^+^ CD29^hi^ PD-L1^+^) and PD-L1*^–^* basal cells (Lin^–^ CD24^+^ CD29^hi^ PD-L1*^–^*) in 3D Matrigel culture. Data were combined from three independent experiments. Data are presented as the mean ± SD. Unpaired *t*-test: **** *P* < 0.0001. Scale bars, 50 μm. **(J)** Representative images of different percentage of fat pad filled with regeneration mammary gland. Scale bars, 1 mm. **(K)** Regeneration efficiency of total (Lin^–^ CD24^+^ CD29^hi^), PD-L1^+^ (Lin^–^ CD24^+^ CD29^hi^ PD-L1^+^) and PD-L1^–^ basal cells (Lin^–^ CD24^+^ CD29^hi^ PD-L1^–^). The mammary outgrowth numbers and sizes (shown as the percentage of fat pad filled) are combined from three independent experiments. The mammary reconstitution unit (MRU) frequency is shown for each group. Student’s *t*-test: **** *P* < 0.0001. PD-L1, programmed cell death ligand 1; MaSCs, mammary stem cells; Procr, protein C receptor; FACS, fluorescence-activated cell sorting; GSEA, gene set enrichment analysis.

Although *PD-L1* mRNA is abundant in many tissues, PD-L1 protein is restricted to immune cells and activated vascular endothelial cells for unknown reasons. We analyzed our RNA-seq data of Procr^+^ MaSCs (Wang et al., 2015) and found that *PD-L1* mRNA was notably enriched in Procr^+^ MaSCs (Figure 1A). We subsequently isolated Procr^+^ and Procr^–^ mammary basal cells (Figure 1B) and quantified *PD-L1* mRNA level by qPCR. The results indicated that *PD-L1* is highly expressed in Procr^+^ mammary basal cells compared to Procr^–^ cells (Figure 1C), which is consistent with the RNA-seq result. To analyze the surface levels of PD-L1, we analyzed various mammary cell populations of adult virgin mice by flow cytometry and identified a small subset of basal cells (2.05 ± 0.89%) with a higher level of PD-L1 expression on the cell surface (Figure 1D); PD-L1 was not detected on luminal cells (Figure 1E). We also found that a subset of stromal cells (3.44 ± 0.87%) expressed PD-L1 (Figure 1F). qPCR analysis of *Procr* expression in PD-L1^+^ and PD-L1^–^ basal cells showed that *Procr* is notably enriched among PD-L1^+^ basal cells (Figure 1G). To globally characterize PD-L1^+^ cells, we used RNA-seq to profile the transcriptomes of PD-L1^+^ basal cells with PD-L1^–^ basal control cells. We then conducted a microarray analysis of PD-L1^+^ basal cells to support direct comparisons against the GSEA signatures of Procr-labeled MaSCs. The results showed that the Procr^+^ MaSC signature genes were significantly upregulated in the transcriptomes of PD-L1^+^ basal cells (Figure 1H). These data suggested that PD-L1^+^ basal cells have MaSCs activities.

### PD-L1^+^ Basal Cells Have the Ability of Colony Formation and Regeneration

To directly investigate the stemness potential of PD-L1^+^ basal cells, we first examined the colony-forming ability of PD-L1^+^ basal cells *in vitro*. PD-L1^+^ basal cells, PD-L1^–^ basal cells, and total basal cells were isolated and cultured in 3D Matrigel. We found that the enrichment of PD-L1^+^ basal cells increased colony-forming efficiency by 5- to 6-folds when compared to the total basal cell group. One colony formed out of 15 plated total basal cells, while 1 colony formed out of 2–3 plated PD-L1^+^ basal cells (Figure 1I). In contrast, PD-L1^–^ basal cells were not able to form typically colonies in Matrigel culture (Figure 1I). Colony sizes were indistinguishable between PD-L1^+^ and total basal cells (Figure 1I). To assess their mammary regeneration capacity, the three groups of isolated cells were transplanted into cleared fat pads. After 8 weeks, PD-L1^+^ basal cells generate the mammary glands more efficiently (repopulating frequency of 1/7.4) than total basal cells (1/64.7) (Figure 1K). Carmine staining analysis showed that the outgrowths displayed normal mammary duct morphology (Figure 1J). In contrast, PD-L1^–^ basal cells showed markedly lower repopulating frequency (1/3228.8) (Figure 1K). Together, these results demonstrate that PD-L1 labels a subset of mammary basal cells with colony formation ability and regeneration capacity, which are typical features of MaSCs.

### *PD-L1^*CreERT2–2A–tdTomato–WERP–pA*^* Knock-In Mouse Recapitulates the *PD-L1* Expression Pattern

**FIGURE 2:**
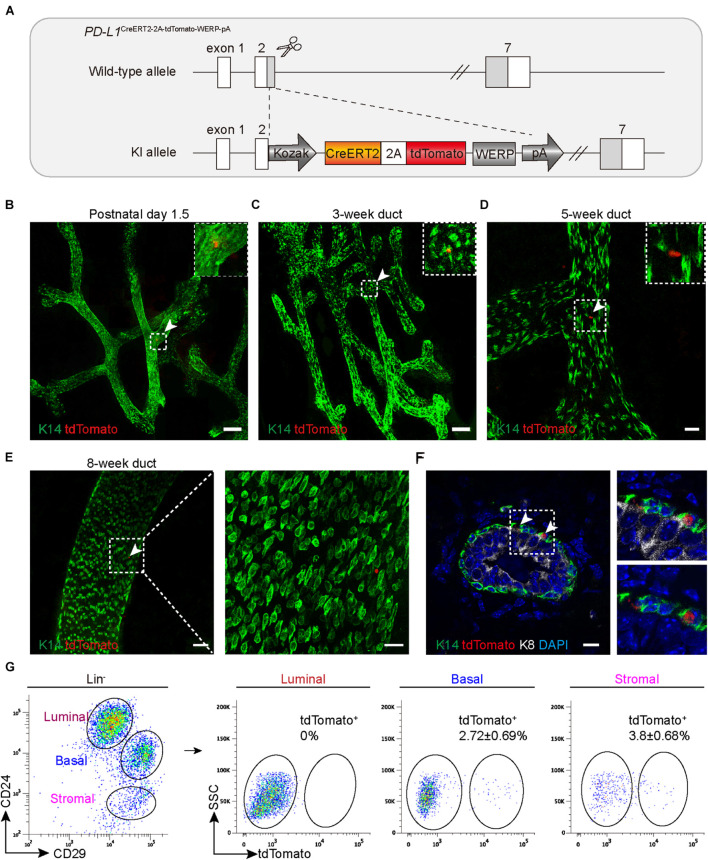
*PD-L1*^CreERT2–2A–tdTomato^ knock-in mouse recapitulates the *PD-L1* expression pattern. **(A)** Targeting strategy to generate *PD-L1*^CreERT2–2A–tdTomato^ knock-in (KI) mouse. **(B–E)** The fourth inguinal mammary glands, harvested from postnatal day 1.5 **(B)**, 3-week-old **(C)**, 5-week-old **(D)**, and 8-week-old, **(E)** *PD-L1*^CreERT2–2A–tdTomato/+^ KI mice, were analyzed by whole-mount confocal imaging. Individual tdTomato^+^ cells were dispersed throughout mammary ducts at all examined stages. A minimum of 50 ducts were analyzed. Scale bars, 100 μm in panels **(B,C)**, 20 μm in panel **(D)**, 50 and 20 μm in panel **(E)**. **(F)** Immunostaining analysis of mammary sections from KI mice. Scale bars, 10 μm. **(G)** FACS analysis indicating that the tdTomato^+^ cells account for 2.72 ± 0.69% of the basal cells and for 3.8 ± 0.68% of stromal cells; *n* = 6 mice. FACS, fluorescence-activated cell sorting; PD-L1, programmed cell death ligand 1.

To better understand the expression pattern and the behavior of PD-L1^+^ basal cells under physiological conditions, we generated PD-L1 report mice in which a CreERT2-2A-tdTomato-WERP-pA cassette was knocked into the second exon of *PD-L1* loci (see the knock-in strategy diagram, Figure 2A). Founder mice with correct genomic modification were confirmed by southern blotting and bred for the subsequent experiments (Supplementary Figure 1a). Whole-mount confocal imaging analysis indicated that tdTomato^+^ (PD-L1^+^) cells were rarely and sparsely positioned in the mammary gland ducts of newborn (postnatal day 1.5) and 3-, 5-, and 8-week-old heterozygous mice (Figures 2B–E). Section imaging of mammary ducts from 8-week-old mice indicated that the tdTomato^+^ cells resided in the basal layer and expressed K14 (Figure 2F). FACS analysis indicated that a small subset of basal cells (2.72 ± 0.69%) was tdTomato^+^, no tdTomato^+^ cells were detected among luminal cells, and a subset of tdTomato^+^ cells (3.8 ± 0.68%) was presented among stromal cells (Figure 2G). These results are consistent with the above results using PD-L1 antibody staining and FACS analysis, supporting that tdTomato reporter recapitulates endogenous expression of *PD-L1*.

### PD-L1^+^ Basal Cells Are Able to Differentiate Into All Mammary Epithelium Cell Lineages

**FIGURE 3:**
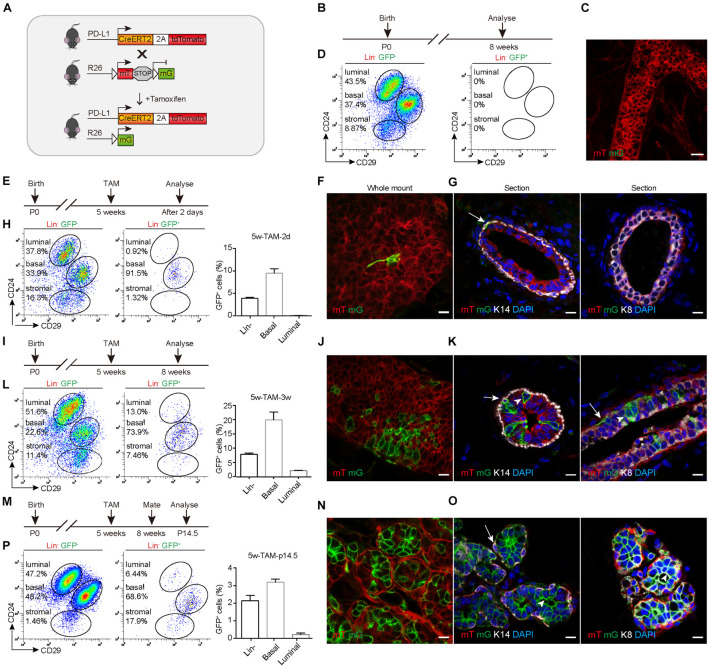
PD-L1^+^ basal cells can produce basal, luminal, and alveolar cells during mammary development. **(A)** Illustration of the lineage-tracing strategy. **(B)** Control, uninduced mice. **(C)** Whole-mount confocal imaging analysis showing that no GFP^+^ cells were present in the mammary ducts of uninduced *PD-L1*^CreERT2–2A–tdTomato/+^; *Rosa26*^mTmG/+^ mice. Scale bars, 20 μm. **(D)** FACS analysis indicating that no GFP^+^ cells were present in the mammary epithelium of uninduced mice. **(E)** Experimental design for the short-term tracing experiments; tamoxifen (TAM) was administered at the pubertal stage (5 weeks old). **(F)** Whole-mount confocal microscope images showing an elongated GFP^+^ basal cell. Scale bars, 10 μm. **(G)** Section imaging indicating the basal location of a GFP^+^ cell. Scale bars, 10 μm. **(H)** FACS analysis indicating that GFP^+^ cells were mainly basal cells at 48 h after tamoxifen administration. **(I–L)** Experimental design for the long-term tracing experiments **(I)**, imaging **(J,K)**, and FACS analysis **(L)** of the distribution of the GFP^+^ cells at 3 weeks after TAM administration. Scale bars, 10 μm. **(M–P)** Experimental design for the long-term tracing experiments **(M),** Imaging **(N,O)**, and FACS analysis **(P)** of the GFP^+^ cell distribution at pregnancy day 14.5. Scale bars, 10 μm. K14^+^ cells are indicated by arrows. K8^+^ cells are indicated by arrowheads; *n* = 5 mice. FACS, fluorescence-activated cell sorting; PD-L1, programmed cell death ligand 1; GFP, green fluorescent protein.

To trace the fate of PD-L1^+^ basal cells, we crossed *PD-L1*^CreERT2–2A–tdTomato–WERP–pA/+^ (*PD-L1*^CreERT2/+^) mice with *Rosa26*^mTmG/+^ (*R26*^mTmG/+^) reporter mice (Muzumdar et al., 2007; Figure 3A) and obtained *PD-L1*^CreERT2/+^ and *R26*^mTmG/+^ reporter mice. After induction by TAM, the PD-L1^+^ cells began to express a green fluorescent protein (GFP) and the offspring of these cells remain GFP^+^, while other non-targeted cells remained red. Using this tracing method, the differentiation potential of PD-L1^+^ basal cells can be obtained by tracking the expression of GFP. No GFP expression was observed in un-induced control mice, as illustrated by whole-mount imaging and FACS analyses (Figures 3B–D). To trace the GFP^+^ cells, we administered TAM to the pubertal reporter mice and analyzed the mice at different time points. Whole-mount confocal imaging analysis of mammary glands after short-time tracing (48 h) showed that single elongated cells were initially labeled with GFP (Figures 3E,F). Immunostaining of tissue sections confirmed that the GFP^+^ cells were K14-labeled basal cells but not K8-labeled luminal cells (Figure 3G and Supplementary Figure 1b), confirming that *PD-L1* is expressed in basal cells under physiological conditions. Quantification of the labeling events by FACS analysis indicated that GFP^+^ cells were mainly basal cells (91.5%) at the beginning of the analysis and GFP^+^ basal cells accounted for about 10% of all basal cells; very few GFP^+^ luminal cells appeared (Figure 3H).

After long-duration tracing (3 weeks), the clonal expansion of GFP^+^ cells was clearly evident in whole-mount confocal images, and we noted that the clones comprised both elongated (basal) and columnar (luminal) cells (Figures 3I,J). Immunostaining in tissue sections confirmed that GFP^+^ cells were present in both basal (K14) and luminal (K8) layers (Figure 3K). Quantification of labeled GFP^+^ cells by FACS analysis showed that 73.9% of GFP^+^ cells were basal cells and 13.0% of GFP^+^ cells were luminal cells. GFP^+^ basal cells accounted for about 20% of all basal cells, GFP^+^ luminal cells account for about 2% of all luminal cells. There were some labeled stromal fibroblasts, which also express PD-L1 (Figure 3L). These results indicated that PD-L1^+^ basal cells produced both basal and luminal cell progenies during the development.

During pregnancy, it was clear that the GFP^+^ cells contributed to alveolus formation, as illustrated by whole-mount confocal imaging analysis (Figures 3M,N). Immunostaining analysis of mammary duct confirmed the presence of GFP^+^ cells in alveolar compartments (Figure 3O). One alveolus could consist solely of GFP^+^ cells or harbor both GFP^+^ and mTomato^+^ cells (Figures 3N,O). Consistently, FACS analysis also indicated that GFP^+^ cells gave rise to basal and luminal cell populations (Figure 3P). Furthermore, we found that GFP^+^ cells were maintained at similar percentages across multiple pregnancies (Supplementary Figures 2a–e), supporting the idea that PD-L1^+^ cells are capable of long-term self-renewal. Together, these results indicated that PD-L1^+^ basal cells can serve as progenitors of all mammary epithelium cell lineages during development and pregnancy.

### *PD-L1* Deficiency Impairs Mammary Gland Development During Puberty and Pregnancy

**FIGURE 4:**
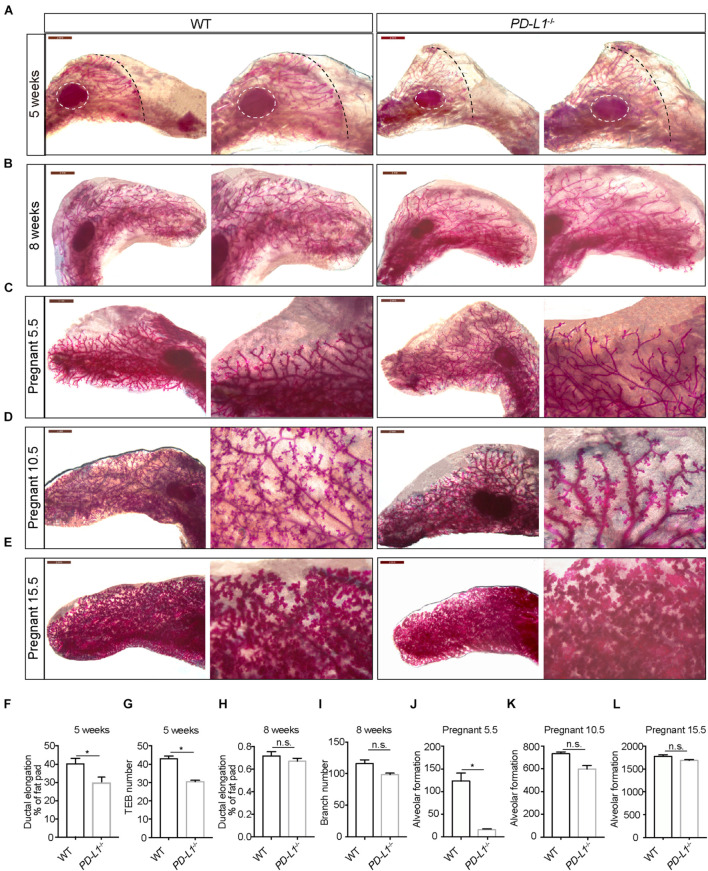
*PD-L1* deficiency impairs mammary gland development during puberty and pregnancy. **(A–E)** Whole-mount imaging (carmine staining) of the mammary epithelium at 5 weeks **(A)**, 8 weeks **(B)**, early pregnancy (Day 5.5) **(C)**, middle pregnancy (Day 10.5) **(D)**, and late pregnancy (Day 15.5) **(E)** of wild-type (WT) and *PD-L1*^–/–^ (homozygous, *PD-L1* deficiency) mice. Scale bars, 2 mm; *n* = 3 mice. **(F)** Quantification of ductal elongation in 5-week-old WT and *PD-L1*^–/–^ mice. Data are presented as mean ± SD. Unpaired *t*-test: * *P* < 0.05. **(G)** Quantification of terminal end bud (TEB) number in 5-week-old WT and *PD-L1*^–/–^ mice. Data are presented as mean ± SD. Unpaired *t*-test: * *P* < 0.05. **(H)** Quantification of ductal elongation in 8-week-old WT and *PD-L1*^–/–^ mice. Data are presented as mean ± SD. Unpaired *t*-test: n.s., not significant. **(I)** Quantification of branch number in 8-week-old WT and *PD-L1*^–/–^ mice. Data are presented as mean ± SD. Unpaired *t*-test: n.s., not significant. **(J)** Quantification of alveolar formation in early pregnant WT and *PD-L1*^–/–^ mice. Data are presented as mean ± SD. Unpaired *t*-test: * *P* < 0.05. **(K)** Quantification of alveolar formation in middle pregnant WT and *PD-L1*^–/–^ mice. Data are presented as mean ± SD. Unpaired *t*-test: n.s., not significant. **(L)** Quantification of alveolar formation in late pregnant WT and *PD-L1*^–/–^ mice. Data are presented as mean ± SD. n.s., not significant; *n* = 5 mice. Both ductal elongation and TEB formation at puberty are reduced in *PD-L1*^–/–^ mice; alveolar formation in early pregnancy is also impaired in *PD-L1*^–/–^ mice. PD-L1, programmed cell death ligand 1.

To investigate whether PD-L1 has a biological function in mammary gland development, we examined the mammary gland phenotypes of *PD-L1*^CreERT2^ homozygous mice (resulting in *PD-L1* deficiency, *PD-L1*^–/–^) at various stages based on carmine staining. At puberty (5 weeks old), compared with the wild-type mice, mammary duct elongation was significantly delayed in *PD-L1* deficient mice, and they displayed decreased formation of TEB (Figures 4A,F,G). In adulthood, there were no significant phenotypes in the *PD-L1*^–/–^ mice, although we did observe a slight reduction in branch number (Figures 4B,H,I). These results suggest that *PD-L1* deficiency delays the development of pubertal mammary glands. During early pregnancy (Day 5.5), the extent of alveolus production was significantly reduced in *PD-L1*^–/–^ mice compared to the wild-type mice (Figures 4C,J). We also detected a mild decrease in alveolar formation in the *PD-L1*^–/–^ mice (Figures 4D,E,K,L) during the middle (Day 10.5) or late (Day 15.5) pregnancy, although it did not reach statistical significance. Together, these results indicated that PD-L1 functions to promote developmentally appropriate mammary duct elongation, TEB formation, and alveolus production.

### *PD-L1* Is Critical for Mammary Regeneration in Transplantation Assay

**FIGURE 5:**
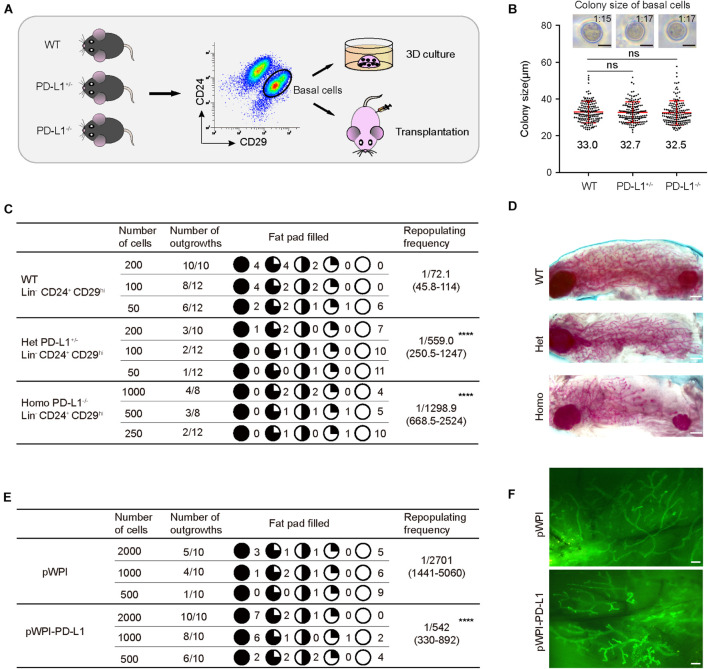
*PD-L1* is critical for mammary regeneration in transplantation. **(A)** Illustration of the 3D culture and transplantation assays. Mammary basal cells were isolated by FACS from WT, PD-L1^+/–^ or PD-L1^–/–^ mice for 3D Matrigel culture or transplantation experiments. **(B)** Colony size of basal cells from WT, PD-L1^+/–^, or PD-L1^–/–^ mice in Matrigel culture. Data are presented as mean ± SD. Scale bars, 50 μm. **(C)** Regeneration frequency of WT, PD-L1^+/–^, or PD-L1^–/–^ basal cells. The mammary outgrowth numbers and sizes (shown as the percentage of fat pad filled) are combined from three independent experiments. The mammary reconstitution unit (MRU) frequency is shown for each group. Student’s *t*-test: **** *P* < 0.0001. **(D)** Representative whole-mount images of outgrowths from transplantation of WT, PD-L1^+/–^, or PD-L1^–/–^ basal cells. Scale bars, 1 mm. **(E)** Regeneration efficiency of pWPI or pWPI-PD-L1 primary mammary cells. The mammary outgrowth numbers and sizes (shown as the percentage of fat pad filled) are combined from three independent experiments. The mammary reconstitution unit (MRU) frequency is shown for each group. Student’s *t*-test: **** *P* < 0.0001. **(F)** Representative whole-mount images of outgrowths from transplantation of pWPI or pWPI-PD-L1 primary mammary cells. Scale bars, 0.5 mm. FACS, fluorescence-activated cell sorting; PD-L1, programmed cell death ligand 1.

Since *PD-L1* deficiency impaired mammary development, we further investigated the functional impact of *PD-L1* on colony formation ability and on the mammary regeneration capacity of mammary basal cells. Briefly, we isolated mammary basal cells from wild-type, PD-L1^+/–^, and PD-L1^–/–^ mice, respectively. The expression of *PD-L1* in basal cells was detected by qPCR (Supplementary Figure 3e). After isolation, the cells were cultured in 3D Matrigel to analyze colony formation ability or transplanted into the cleared fat pads of recipient mice to analyze regeneration capacity (Figure 5A).

In 3D Matrigel culture assay, we found that colony size of wild-type basal cells is 33 ± 1.4 μm and 1 colony formed out of about 15 plated total basal cells; colony size of PD-L1^+/–^ basal cells is 32.7 ± 1.5 μm and 1 colony formed out of about 17 plated total basal cells; and colony size of PD-L1^–/–^ basal cells is 32.5 ± 1.8 μm and 1 colony formed out of about 17 plated total basal cells (Figure 5B). These results demonstrated that PD-L1 deficiency does not impair the colony formation ability of mammary basal cells *in vitro*.

In the transplantation assays, we found that knockdown (PD-L1^+/–^ basal cells) and knockout (PD-L1^–/–^ basal cells) of *PD-L1* resulted in a significant attenuation in the regeneration capacity of mammary basal cells, as evidenced by decreased repopulating frequency (wild-type basal cells: 1/72.1; PD-L1^+/–^ basal cells: 1:559.0; PD-L1^–/–^ basal cells: 1:1298.9; Figure 5C). Whereas, we observed that there were no significant phenotypes in the regenerate outgrowths of PD-L1^+/–^ and PD-L1^–/–^ basal cells, as evidenced by normal mammary duct growth (Figure 5D).

To further analyze the role of PD-L1 in mammary regeneration, we used a gain of function method. We overexpressed *PD-L1* in basal cells using *PD-L1* expressing lentiviral vector (named pWPI-PD-L1) and compared the regeneration capacity with that of control cells containing empty vector (named pWPI). *PD-L1* overexpression was confirmed by qPCR and western blot analysis (Supplementary Figure 3a). We found that overexpression of *PD-L1* promoted mammary regeneration capacity (Figure 5E: pWPI: 1:2701; pWPI-PD-L1: 1:542) without any obvious alteration of the morphology of regenerated mammary glands (Figure 5F). Together, these results demonstrated a functional impact of *PD-L1* in promoting the regenerative capacity of mammary basal cells.

## Discussion

Here, we discovered previously unidentified PD-L1^+^ mammary basal cells and showed how these function as progenitors of all mammary epithelium cells: lineage tracing demonstrated their capacity to give rise to new basal, luminal, and alveolar cells during mammary development. Specific deletion of *PD-L1* delayed mammary gland development, with phenotypes, such as disruption of TEB and alveolar formation, and dysregulated duct elongation. Additionally, our results in transplantation assays support that expression of *PD-L1* regulates the efficiency of mammary regeneration.

Likely, owing to the extremely low PD-L1 protein expression level in most tissues and organs under physiological conditions, few studies have focused on identifying and functionally characterizing subsets of PD-L1^+^ cells. Related results from the literature include the notable findings from studies of tumors that *PD-L1* expression may be clustered rather than diffuse (Dong et al., 2002; Taube et al., 2012). Further, recent studies have demonstrated that there is a PD-L1^+^ NK cell subset involved in anti-PD cancer therapy against PD-L1^–^ tumors (Dong et al., 2019), and another subset of cells in healthy islets was found to express *PD-L1* and overexpression of *PD-L1* can protect human islet-like organoid transplantation from immune rejection (Yoshihara et al., 2020). Our work in the present study showed that PD-L1^+^ basal cells of the mammary epithelium exhibit high regeneration frequency, and we found that overexpression of *PD-L1* promoted mammary regeneration frequency in transplantation assays, indicating that PD-L1^+^ cell subsets in tissues and organs can exert functions related to tissue damage repair and tissue regeneration. Thus, our study further strengthens the notion that *PD-L1* expression can be applied to promote organ regeneration.

Many efforts have been invested in delineating the lineage relationship(s) among mammary epithelial cells, which has yielded discoveries, such as the definition of Procr (Wang et al., 2015), Bcl11b (Cai et al., 2017), and Dll1 (Chakrabarti et al., 2018) as markers of MaSCs. Procr is expressed in mammary basal and stromal cells, but not luminal cells, and it is understood to label multipotent and activated MaSCs. Dll1 is specifically expressed in mammary basal cells, labeling bipotent MaSCs; this protein has been shown to mediate cross-talk between MaSCs and macrophages in the mammary gland. Bcl11b is specifically expressed in basal cells, labeling a quiescent population of MaSCs located at the basal-luminal interface, and Bcl11b is necessary for long-term maintenance of the mammary gland. Our study adds PD-L1 to the list of markers for MaSCs. Of note, there are many similarities between PD-L1^+^ basal cells and Procr^+^ basal cells: First, both PD-L1^+^ basal cells and Procr^+^ basal cells have a high regenerative capacity and can differentiate into all lineages of the mammary epithelium cells. Second, both PD-L1^+^ basal cells and Procr^+^ basal cells exhibit EMT signatures. Third, GSEA analysis showed that gene signatures of Procr^+^ MaSC were enriched in PD-L1^+^ basal cells. Despite these similar characteristics, there is an important difference that bears emphasis: there is only about 30–40% overlap between PD-L1^+^ and Procr^+^ basal cells (Supplementary Figure 3b), suggesting that both Procr and PD-L1 may only label partial of MaSCs or they did not label pure MaSCs. Besides, the proportion of Procr^+^ basal cells remains unchanged among mammary development (Wang et al., 2015), but the proportion of PD-L1^+^ cells indicated a significant variation. Specifically, in adolescence, PD-L1-labeled basal cells were about 4.77% (4.77 ± 0.98), in adulthood, PD-L1-labeled basal cells were about 2.04% (2.04 ± 0.91), and during pregnancy and lactation, the proportion of PD-L1-labeled basal cells was very low (less than 0.5%; Supplementary Figures 4a–d). These different distributions between the two groups of basal cells during mammary development suggest that there are functional differences between PD-L1^+^ basal cells and Procr^+^ basal cells. The relationship between these cell populations needs to be further studied. Considering the known function mediated by PD-L1/PD-1 signal, we speculate that PD-L1 may be involved in mediating the interaction between MaSCs and T cells in the mammary gland.

Endogenous PD-L1 expression can be induced or maintained by many cytokines, of which interferon-γ (IFN-γ) is currently thought to be the most potent (Sun et al., 2018). Immune-induced tumor PD-L1 expression is considered to be an adaptive resistance mechanism by tumor cells in response to immune challenge, and PD-L1 expression can also be regulated by intrinsic oncogenic pathways, microRNA, genetic alterations, and post-translational regulation (Zou et al., 2016; Sun et al., 2018). These regulatory mechanisms have mainly been discovered in the context of immune cells and in many cancers. Wnt/ß-catenin signaling is known to regulate mammary gland development (Zeng and Nusse, 2010; van Amerongen et al., 2012). We assessed the effects of Wnt/ß-catenin signaling on PD-L1 expression in the present study. We isolated mammary basal cells and cultured them in 3D Matrigel in the presence of two Wnt/ß-catenin signaling activators: the ligand Wnt3a or the small molecule CHIR99021 (CHIR). qPCR analysis indicated that PD-L1 expression was significantly upregulated in the presence of Wnt3a or CHIR (Supplementary Figures 3c,d). These results demonstrated that *PD-L1* expression is upregulated by continuous activation of Wnt/ß-catenin signaling in mammary basal cells under 3D Matrigel culture. To investigate whether PD-L1 expression in mammary glands is regulated by Wnt signal, we utilized *MMTV-Wnt1* transgenic mice (Tsukamoto et al., 1988) to continuously activate the Wnt/ß-catenin signal *in vivo*. FACS analysis indicated that the proportion of PD-L1^+^ basal cells in mammary glands of MMTV-Wnt1 mice was changed significantly: at puberty (5 weeks), PD-L1-labeled basal cells were about 0.47 ± 0.09% of Supplementary Figure 5d; in adulthood (10 weeks), PD-L1 labeled about 0.68 ± 0.13% of basal cells (Supplementary Figure 5e); and in 16 weeks old, PD-L1 labeled about 0.82 ± 0.11% of basal cells (Supplementary Figure 5f). Compared with wild-type mice (Supplementary Figures 5a–c). The protein level of PD-L1 was upregulated in *MMTV-Wnt1* transgenic mice, suggesting that PD-L1 protein expression is upregulated by the Wnt/ß-catenin signal. Considering that the Wnt/ß-catenin signaling pathway is activated during the development of many tissues and organs, this may explain that *PD-L1* mRNA is abundant in most tissues and organs. However, the expression of PD-L1 protein needs more inducers.

Indeed, the mechanism by which PD-L1 contributes to stem cell function remains elusive. Many studies have focused on the relationship between PD-L1 and CSCs: PD-L1 inhibits T cell function through the PD-1 receptor, which is very important for tumor cells to escape immune surveillance (Wei et al., 2019). In triple-negative breast cancer, Wnt signaling can upregulate PD-L1 expression and significantly enrich the signal related to immunity and cancer stemness (Castagnoli et al., 2019). Similarly, there is a bidirectional effect between EMT status and PD-L1 expression in breast cancer (Mani et al., 2008; Alsuliman et al., 2015). In our study, we found that EMT-related genes were enriched in PD-L1^+^ basal cells (Supplementary Figures 6a–c), which further proved that PD-L1^+^ basal cells might have the characteristics of breast CSCs. In addition, we speculate that PD-L1 may be involved in regulating the interaction between MaSCs and T cells, thus to ensure normal mammary gland development. Here, our study provides a novel insight into the expression and physiological functions of PD-L1 in mammary development, implicating that breast CSCs arise from PD-L1^+^ stem cells are immune privileged because of PD-L1. However, the other side of the sword is that PD-L1 can also protect the tumor (stem) cells from immune attack. Accordingly, the PD-L1^+^ cells we discovered in the present study represent an excellent experimental system for elucidating this hypothetical mechanism.

In conclusion, we have identified and characterized a PD-L1^+^ subset of mammary basal cells, suggesting that PD-L1 is involved in more physiological processes than its previously known functions as an immune mediator. Furthermore, our results strengthened the correlation between *PD-L1* expression and the Wnt/ß-catenin signaling pathway. Together, our study provides a new conceptual space for studying the function of *PD-L1* in organogenesis and exploring its potential application in regenerative medicine.

## Limitation of Study

Although our study identified and characterized the PD-L1^+^ subset of mammary basal cells, it has some clear limitations. First, PD-L1 is a well-known immune regulatory factor, as a cell surface marker of mammary stem/progenitor cells, it may involve mediating the cross-talk between stem cells and immune cells, especially T cells, which is not investigated in the present study. Second, our current research cannot explain the physiological significance of Wnt/ß-catenin signaling on regulation of *PD-L1* transcription. Regarding the constitutive activation of Wnt/ß-catenin signaling that can induce the initiation of breast cancer, there may be a correlation between the initiation of breast cancer and the regulation of *PD-L1* transcription. Third, there have been many studies on the identification of MaSCs, and various molecular markers have been discovered. The current research has undoubtedly increased the complexity of this field. What kind of internal relationship exists among these subsets of cells is still unknown. Perhaps single-cell profiling datasets can be used to further explore it.

## Data Availability Statement

The datasets presented in this study can be found in online repositories. The names of the repository/repositories and accession number(s) can be found below: https://www.ncbi.nlm.nih.gov/, GSE166131.

## Ethics Statement

The animal study was reviewed and approved by Animal Care and Use Committee of Wuhan University.

## Author Contributions

RW and CC designed the experiments. RW and FH performed the experiments. WW, YZ, ZY, and LY analyzed the data. RW, JH, and CC wrote the manuscript. All authors read and approved the final manuscript.

## Conflict of Interest

JH was employed by the company ‘Shenzhen Beike Biotechnology Co., Ltd’. The remaining authors declare that the research was conducted in the absence of any commercial or financial relationships that could be construed as a potential conflict of interest.

## Publisher’s Note

All claims expressed in this article are solely those of the authors and do not necessarily represent those of their affiliated organizations, or those of the publisher, the editors and the reviewers. Any product that may be evaluated in this article, or claim that may be made by its manufacturer, is not guaranteed or endorsed by the publisher.

## Supplementary Material

Supplementary Figure 1 Genotyping of *PD-L1*^CreERT2–2A–tdTomato–WERP–pA^ knock-in mouse, and tracing of *PD-L1* expressing cells. **(a)** Southern blot analysis showing the genotype of *PD-L1*^CreERT2–2A–tdTomato–WERP–pA^ knock-in mouse. **(b)** Section imaging showed mT, mG, K14, DAPI of the ducts in Figure 3G, respectively. Scale bars, 10 μm. PD-L1, programmed cell death ligand 1.

Supplementary Figure 2 PD-L1^+^ cells maintain multipotency of MaSCs beyond multiple rounds of pregnancy. **(a)** Tamoxifen was administered in 5-week-old *PD-L1*^CreERT2–2A–tdTomato/+^; *Rosa26*^mTmG/+^ mice. Labeled cell contribution was analyzed as illustrated in panel **(a)**. **(b,c)** FACS analysis of *PD-L1*^CreERT2–2A–tdTomato/+^; *Rosa26*^mTmG/+^ mice, indicating that GFP^+^ cells were distributed in both the basal and luminal layer in the second pregnancy and the third pregnancy; *n* = 3 mice. **(d)** Quantification of FACS analysis of *PD-L1^CreERT2–2A–tdTomato/+^; Rosa26^mTmG/+^* mice, indicating that GFP^+^ cells were distributed in both the basal (majority) and luminal layer (minority) in the second pregnancy; *n* = 3 mice. **(e)** Quantification of GFP^+^ cells, indicating no difference in the percentage of GFP^+^ basal cells between nulliparous mice and multiparous mice that have gone through three complete cycles of pregnancy and involution; *n* = 3 mice. FACS, fluorescence-activated cell sorting; PD-L1, programmed cell death ligand 1; GFP, green fluorescent protein.

Supplementary Figure 3 *PD-L1* expression under various conditions. **(a)** qPCR and western blot analysis validating the overexpression efficiency of *PD-L1*. **(b)** FACS analysis of the overlap of PD-L1^+^ and Procr^+^ subpopulations of mammary basal cells. **(c)** qPCR quantification of *PD-L1* mRNA levels in cultured basal cells (6 and 10 days) in the presence of purified Wnt3a protein (ligand of Wnt/ß-catenin signaling); *Axin2* expression was used to show the activation of Wnt/ß-catenin signaling. Data are presented as the mean ± SD. Unpaired *t*-test: ^∗^ *P* < 0.05,^∗∗^ *P* < 0.01. n.s., not significant. **(d)** qPCR quantification of *PD-L1* mRNA levels in cultured basal cells in the presence of CHIR (activator of Wnt/ß-catenin signaling). *Axin2* expression was used to show the activation of Wnt/ß-catenin signaling. Data are presented as the mean ± SD. Unpaired *t*-test: ^∗∗^ *P* < 0.01. **(e)** qPCR quantification of *PD-L1* mRNA levels in basal cells from WT, PD-L1^+/^*^–^* and PD-L1^–/–^ mice, respectively. Data are presented as the mean ± SD. Unpaired *t*-test: ^****^ *P* < 0.0001. n.s., not significant. FACS, fluorescence-activated cell sorting; PD-L1, programmed cell death ligand 1; Procr^+^, protein C receptor.

Supplementary Figure 4 PD-L1 expression pattern during various mammary development stages of wild-type mice. **(a)** FACS analysis of PD-L1^+^ basal cells in mammary glands of pubertal (5–6 weeks) wild-type mice; *n* = 3 mice. **(b)** FACS analysis of PD-L1^+^ basal cells in mammary glands of adult (8–12 weeks) wild-type mice; *n* = 3 mice. **(c)** FACS analysis of PD-L1^+^ basal cells in mammary glands of pregnant wild-type mice; *n* = 3 mice. **(d)** FACS analysis of PD-L1^+^ basal cells in mammary glands of lactating wild-type mice; *n* = 3 mice. FACS, fluorescence-activated cell sorting; PD-L1, programmed cell death ligand 1.

Supplementary Figure 5 PD-L1 transcription is regulated by Wnt/ß-catenin signaling. **(a–c)** FACS analysis of PD-L1 expression in mammary glands of 5-, 10-, and 16-week-old wild-type mice. All mice are FVB/N genetic background. The proportion of PD-L1^+^ basal cells were analyzed; *n* = 3 mice. **(d–f)** FACS analysis of PD-L1 expression in mammary glands of 5-, 10-, and 16-week-old *MMTV-Wnt1* transgenic mice. All mice are FVB/N genetic background. The proportion of PD-L1^+^ basal cells were analyzed; *n* = 3 mice. FACS, fluorescence-activated cell sorting; PD-L1, programmed cell death ligand 1.

Supplementary Figure 6 PD-L1^+^ mammary basal cells enrich EMT-related genes. **(a,b)** Heat map analysis showed that expression of EMT-related **(b)** genes was enriched in PD-L1^+^ basal cells. **(c)** qPCR analysis of EMT-related genes, which shows elevated expression in PD-L1^+^ basal cells compared to PD-L1^–^ basal cells. Data are presented as the mean ± SD. Unpaired *t*-test: ^∗^ *P* < 0.05; *n* = 3. PD-L1, programmed cell death ligand 1; ENT, epithelial-to-mesenchymal transition.
