# Supplementary figures and images for: Programmed Cell Death Ligand 1 Is Enriched in Mammary Stem Cells and Promotes Mammary Development and Regeneration
Source: Front Cell Dev Biol. 2021 Nov 5;9:772669. doi: 10.3389/fcell.2021.772669 (PMC8602569; doi:10.3389/fcell.2021.772669)

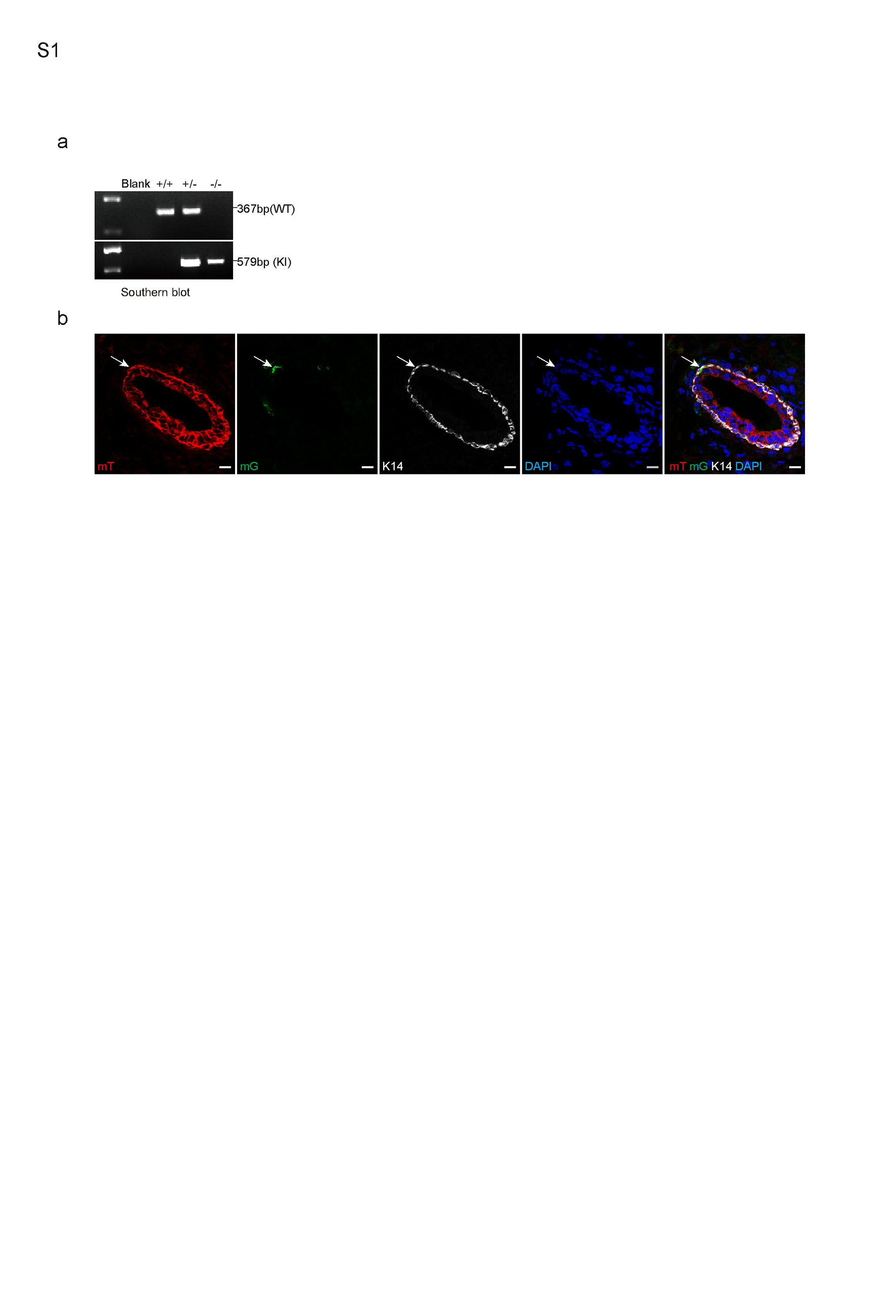

Supplement: Supplementary Figure 1 — Genotyping of PD-L1CreERT2–2A–tdTomato–WERP–pA knock-in mouse, and tracing of PD-L1 expressing cells. (a) Southern blot analysis showing the genotype of PD-L1CreERT2–2A–tdTomato–WERP–pA knock-in mouse. (b) Section imaging showed mT, mG, K14, DAPI of the ducts in Figure 3G, respectively. Scale bars, 10 μm. PD-L1, programmed cell death ligand 1. [file Image_1.TIF]

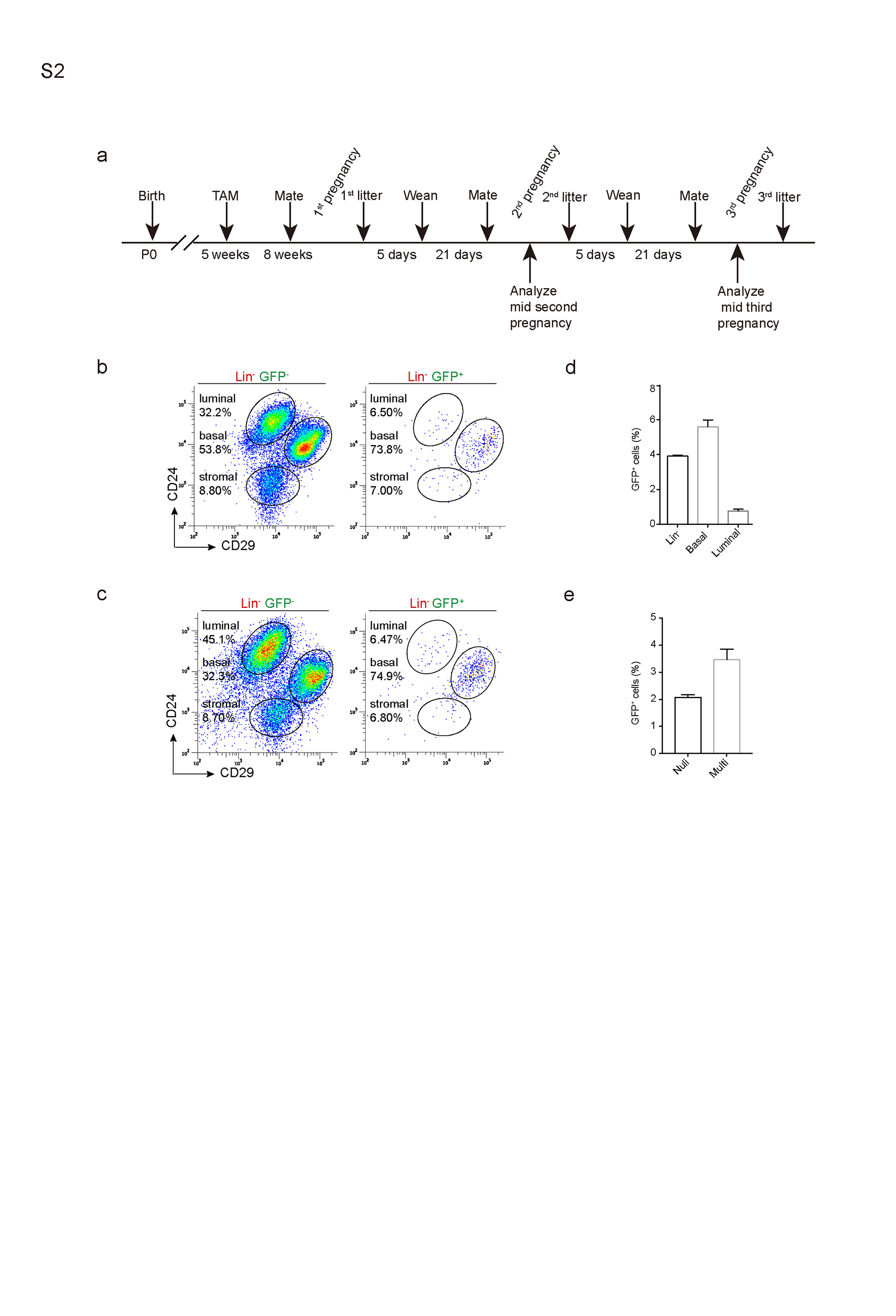

Supplement: Supplementary Figure 2 — PD-L1+ cells maintain multipotency of MaSCs beyond multiple rounds of pregnancy. (a) Tamoxifen was administered in 5-week-old PD-L1CreERT2–2A–tdTomato/+; Rosa26mTmG/+ mice. Labeled cell contribution was analyzed as illustrated in panel (a). (b,c) FACS analysis of PD-L1CreERT2–2A–tdTomato/+; Rosa26mTmG/+ mice, indicating that GFP+ cells were distributed in both the basal and luminal layer in the second pregnancy and the third pregnancy; n = 3 mice. (d) Quantification of FACS analysis of PD-L1CreERT2–2A–tdTomato/+; Rosa26mTmG/+ mice, indicating that GFP+ cells were distributed in both the basal (majority) and luminal layer (minority) in the second pregnancy; n = 3 mice. (e) Quantification of GFP+ cells, indicating no difference in the percentage of GFP+ basal cells between nulliparous mice and multiparous mice that have gone through three complete cycles of pregnancy and involution; n = 3 mice. FACS, fluorescence-activated cell sorting; PD-L1, programmed cell death ligand 1; GFP, green fluorescent protein. [file Image_2.TIF]

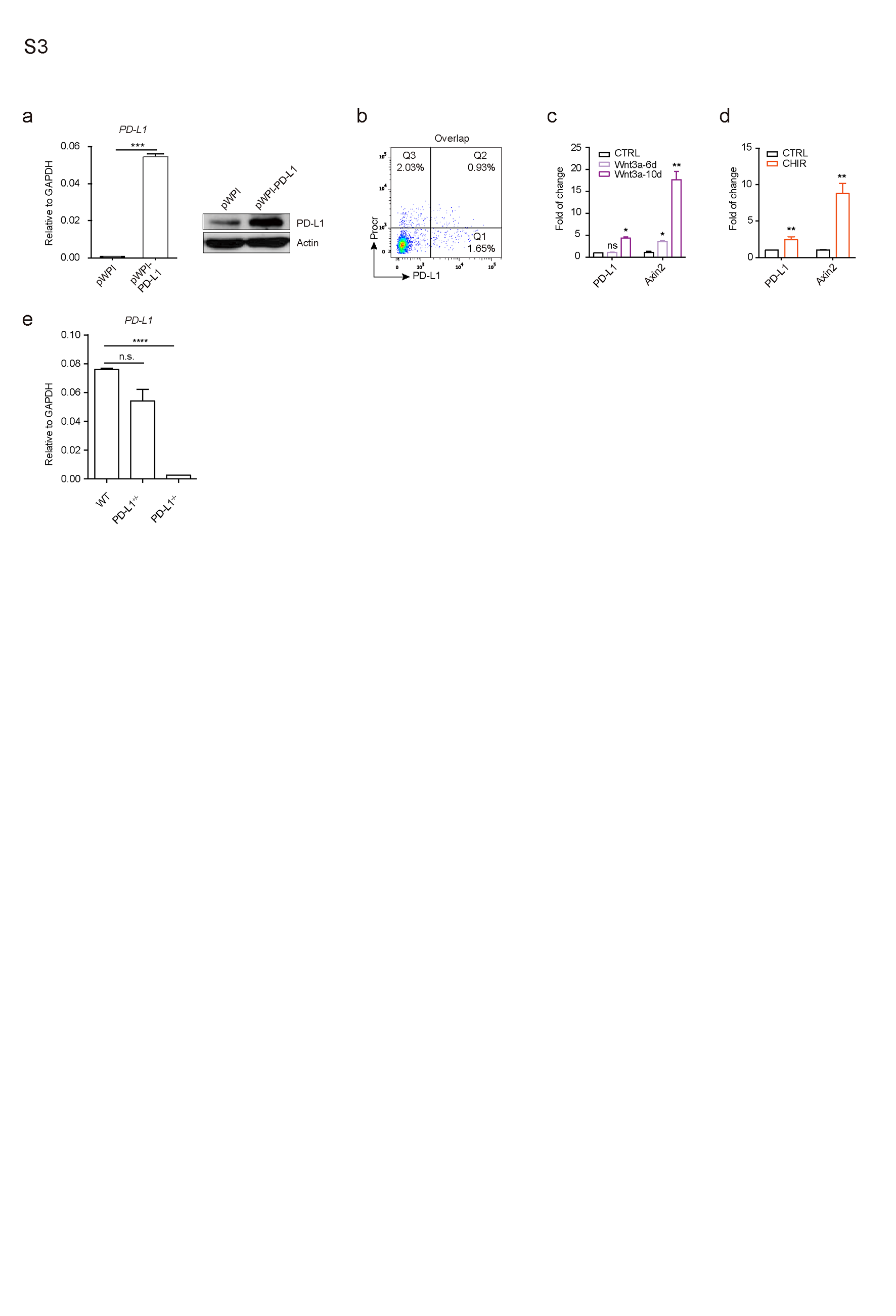

Supplement: Supplementary Figure 3 — PD-L1 expression under various conditions. (a) qPCR and western blot analysis validating the overexpression efficiency of PD-L1. (b) FACS analysis of the overlap of PD-L1+ and Procr+ subpopulations of mammary basal cells. (c) qPCR quantification of PD-L1 mRNA levels in cultured basal cells (6 and 10 days) in the presence of purified Wnt3a protein (ligand of Wnt/ß-catenin signaling); Axin2 expression was used to show the activation of Wnt/ß-catenin signaling. Data are presented as the mean ± SD. Unpaired t-test: ∗ P < 0.05,∗∗ P < 0.01. n.s., not significant. (d) qPCR quantification of PD-L1 mRNA levels in cultured basal cells in the presence of CHIR (activator of Wnt/ß-catenin signaling). Axin2 expression was used to show the activation of Wnt/ß-catenin signaling. Data are presented as the mean ± SD. Unpaired t-test: ∗∗ P < 0.01. (e) qPCR quantification of PD-L1 mRNA levels in basal cells from WT, PD-L1+/– and PD-L1–/– mice, respectively. Data are presented as the mean ± SD. Unpaired t-test: **** P < 0.0001. n.s., not significant. FACS, fluorescence-activated cell sorting; PD-L1, programmed cell death ligand 1; Procr+, protein C receptor. [file Image_3.TIF]

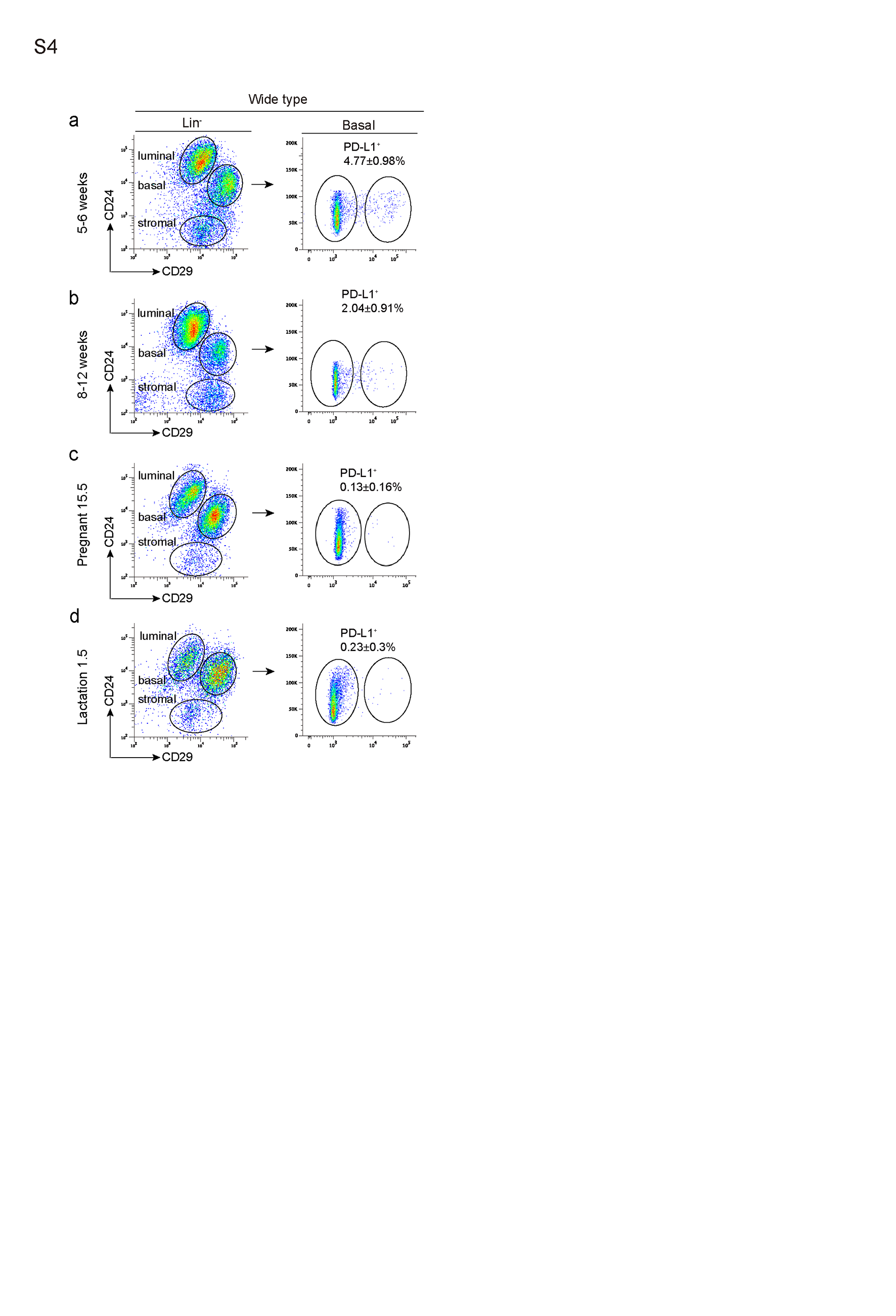

Supplement: Supplementary Figure 4 — PD-L1 expression pattern during various mammary development stages of wild-type mice. (a) FACS analysis of PD-L1+ basal cells in mammary glands of pubertal (5–6 weeks) wild-type mice; n = 3 mice. (b) FACS analysis of PD-L1+ basal cells in mammary glands of adult (8–12 weeks) wild-type mice; n = 3 mice. (c) FACS analysis of PD-L1+ basal cells in mammary glands of pregnant wild-type mice; n = 3 mice. (d) FACS analysis of PD-L1+ basal cells in mammary glands of lactating wild-type mice; n = 3 mice. FACS, fluorescence-activated cell sorting; PD-L1, programmed cell death ligand 1. [file Image_4.TIF]

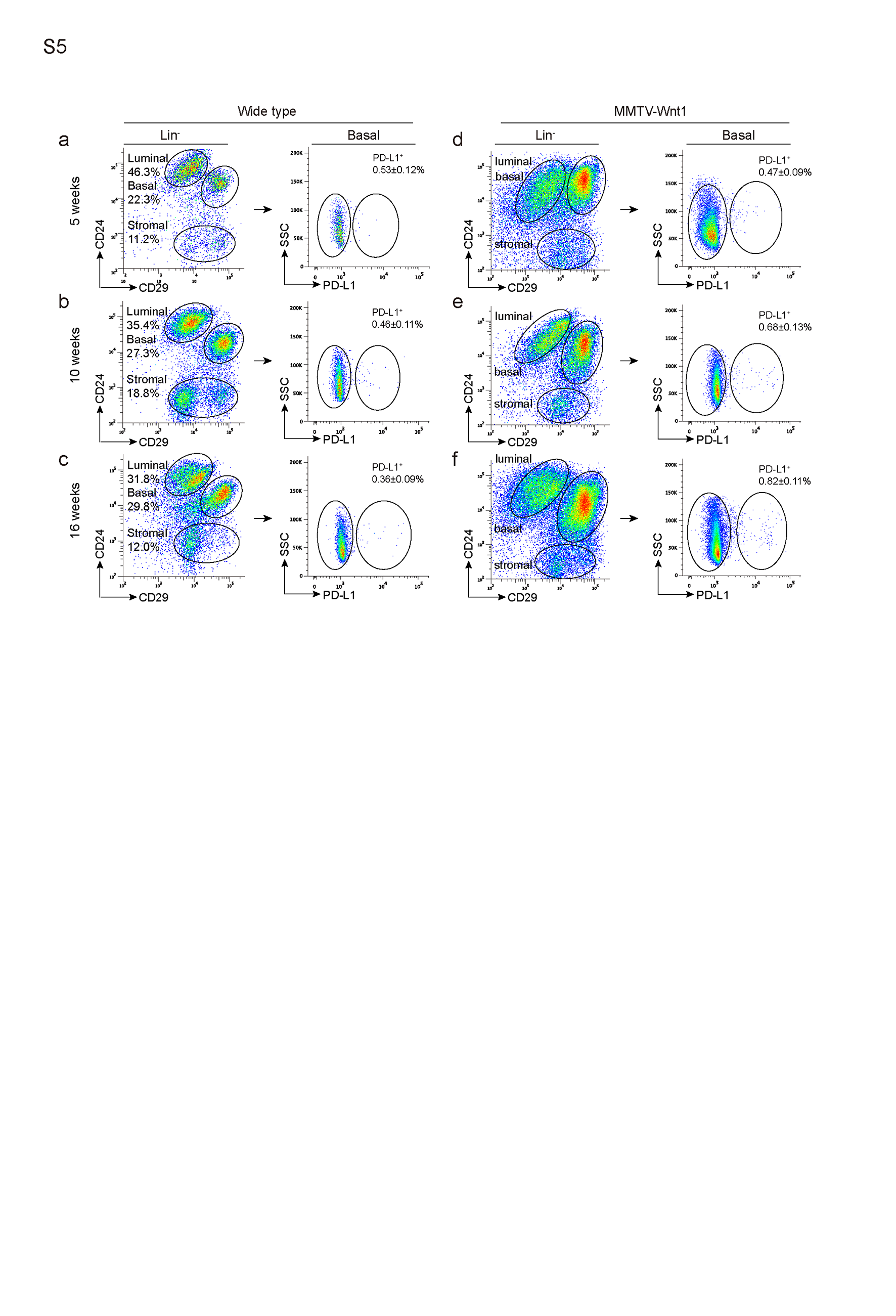

Supplement: Supplementary Figure 5 — PD-L1 transcription is regulated by Wnt/ß-catenin signaling. (a–c) FACS analysis of PD-L1 expression in mammary glands of 5-, 10-, and 16-week-old wild-type mice. All mice are FVB/N genetic background. The proportion of PD-L1+ basal cells were analyzed; n = 3 mice. (d–f) FACS analysis of PD-L1 expression in mammary glands of 5-, 10-, and 16-week-old MMTV-Wnt1 transgenic mice. All mice are FVB/N genetic background. The proportion of PD-L1+ basal cells were analyzed; n = 3 mice. FACS, fluorescence-activated cell sorting; PD-L1, programmed cell death ligand 1. [file Image_5.TIF]

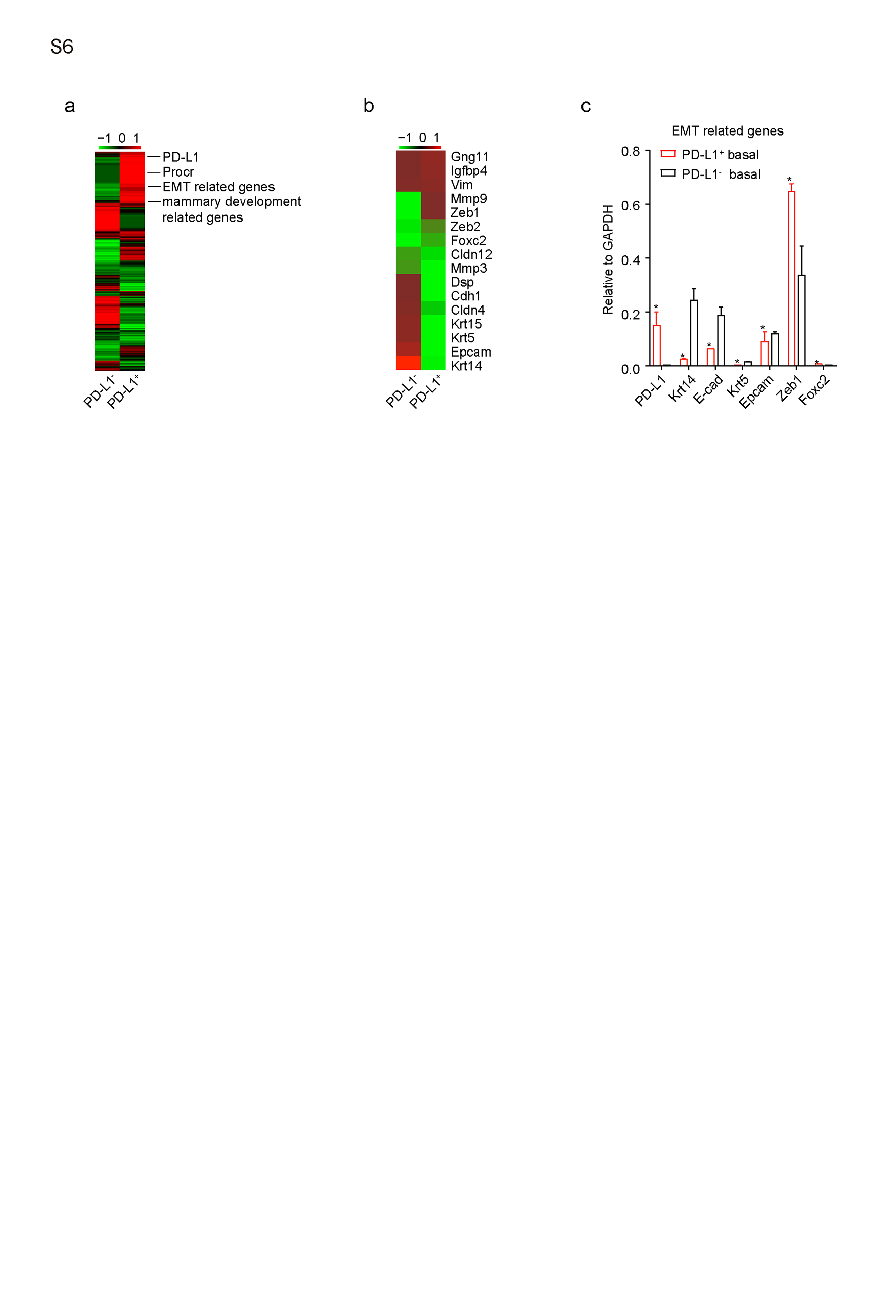

Supplement: Supplementary Figure 6 — PD-L1+ mammary basal cells enrich EMT-related genes. (a,b) Heat map analysis showed that expression of EMT-related (b) genes was enriched in PD-L1+ basal cells. (c) qPCR analysis of EMT-related genes, which shows elevated expression in PD-L1+ basal cells compared to PD-L1– basal cells. Data are presented as the mean ± SD. Unpaired t-test: ∗ P < 0.05; n = 3. PD-L1, programmed cell death ligand 1; ENT, epithelial-to-mesenchymal transition. [file Image_6.TIF]
